# Intestinal organoid modeling: bridging the gap from experimental model to clinical translation

**DOI:** 10.3389/fonc.2024.1334631

**Published:** 2024-03-01

**Authors:** Taotao Liu, Xiaoqi Li, Hao Li, Jingjing Qin, Hui Xu, Jun Wen, Yaqin He, Cao Zhang

**Affiliations:** ^1^ School of Clinical Medicine, Ningxia Medical University, Yin Chuan, Ningxia, China; ^2^ Department of Gastrointestinal Surgery, Affiliated Hospital of Ningxia Medical University, Yin Chuan, Ningxia, China; ^3^ School of Clinical Medicine, Shandong Second Medical University, Weifang, Shandong, China; ^4^ Department of Anesthesiology and Surgery, Gansu Provincial People's Hospital, Lan Zhou, Gansu, China

**Keywords:** intestinal organoids, intestinal tumor organoids, 3D culture, culture environment, precision medicine

## Abstract

The 3D culture of intestinal organoids entails embedding isolated intestinal crypts and bone marrow mesenchymal stem cells within a growth factor-enriched matrix gel. This process leads to the formation of hollow microspheres with structures resembling intestinal epithelial cells, which are referred to as intestinal organoids. These structures encompass various functional epithelial cell types found in the small intestine and closely mimic the organizational patterns of the small intestine, earning them the name “mini-intestines”. Intestinal tumors are prevalent within the digestive system and represent a significant menace to human health. Through the application of 3D culture technology, miniature colorectal organs can be cultivated to retain the genetic characteristics of the primary tumor. This innovation offers novel prospects for individualized treatments among patients with intestinal tumors. Presently established libraries of patient-derived organoids serve as potent tools for conducting comprehensive investigations into tissue functionality, developmental processes, tumorigenesis, and the pathobiology of cancer. This review explores the origins of intestinal organoids, their culturing environments, and their advancements in the realm of precision medicine. It also addresses the current challenges and outlines future prospects for development.

## Graphical abstract

A summary of the intestinal organoid culturing process and its associated applications is provided.

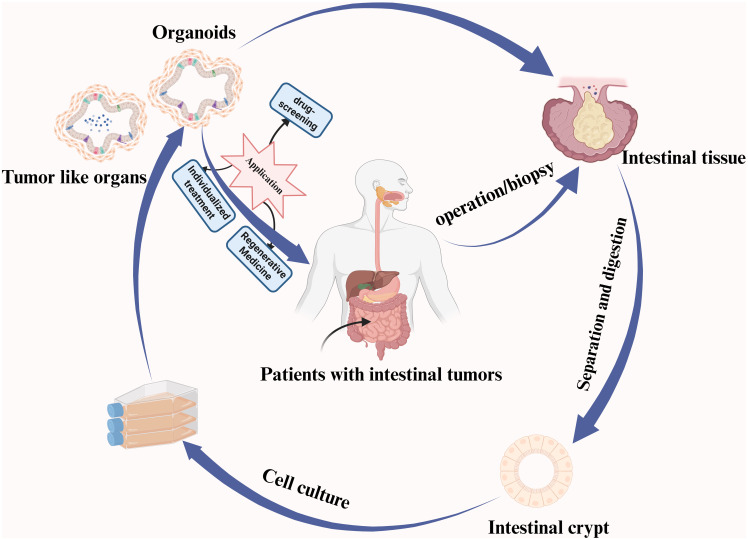

## 1 Introduction

At present, traditional two-dimensional cancer cell lines continue to serve as the cornerstone of human research. However, only a limited number of cancer cells can be successfully cultured, and due to their significant adaptation to the culture medium, most tumor cell lines no longer accurately reflect the heterogeneity and genetic traits of the original tumor tissue. In contrast, patient-derived tumor xenografts (PDTX) maintain histological and genomic aberration patterns more faithfully to those of parental tumors. Nevertheless, the prolonged time and elevated costs associated with their establishment restrict their utility in cancer research (1). Organoids, a relatively recent three-dimensional culture system, have opened new possibilities in intestinal tumor research. These structures are synthetic three-dimensional cellular assemblies primarily driven by stem cells or pluripotent stem cells. They are typically self-assembled and engineered to replicate the structure and function of human organs. Research in this field has revealed that organoids are capable of preserving the heterogeneity and genetic characteristics of the originating cancerous tissue (2–4).

The recent establishment of extensive repositories housing live patient-derived cancer organoid (PDO) libraries offers fresh avenues for in-depth analysis of cancer biology and clinical translational applications (5). Intestinal stem cells are gaining increasing prominence, primarily employed for investigating the regeneration of intestinal injuries and various conditions like intestinal tumors. Currently, the widely recognized marker for intestinal stem cells is Lgr5, which plays a pivotal role in preserving the integrity of the intestinal epithelium (6). As per pertinent research, culturing isolated intestinal stem cells and intestinal crypts in a matrix gel with suitable growth factors through a 3D approach can lead to the development of intestinal organoids with an internal structure closely resembling that of the intact intestinal epithelium (7). Intestinal organoids offer substantial advantages, including a high success rate in culture, a brief culture period, a close resemblance to primary tissue characteristics, and ease of establishing malignant tumor models (8). Moreover, organoids can be generated from samples obtained through surgical procedures or biopsies, allowing for the creation of individual-specific intestinal models with potential applications in precision medicine. Research in this domain is highly promising, and diverse facets of organoid studies are advancing swiftly, contributing to the evolution of this emerging field. Within this review, we delve into the origins and cultivation environments of intestinal organoids, chart the advancements in their application within the realm of precision medicine, and address both the current challenges and future prospects for further development.

## 2 Extracellular matrix supporting intestinal tumor organoid culture

Similar to the cultivation of common organs, the extracellular matrix (ECM) plays a pivotal role in providing essential support for the cultivation of intestinal tumor-like organoids (10, 11). Organoids encompass various extracellular matrix types, broadly classified into two categories: naturally occurring and synthetic. Each category comprises distinct protein sets and molecules, each fulfilling diverse roles in facilitating cell growth, morphology, and functionality (**Table 1**) (9, 12). Matrigel originates from the natural basement membrane matrix of mouse tumors and comprises an array of matrix proteins like collagen and proteoglycans, facilitating the attachment and proliferation of intestinal cells while fostering the formation and growth of organoids. Additionally, Matrigel encompasses diverse growth factors and extracellular signaling molecules, mirroring the microenvironment of intestinal tissues, crucial for fostering optimal conditions supporting cell growth and functional development (13). Collagen, a primary connective tissue protein abundant in intestinal tissues, establishes the essential environment necessary for cell attachment and growth. When integrated into organoid culture, collagen serves to replicate the natural tissue milieu, fostering cell attachment, proliferation, and growth. Furthermore, its presence aids in guiding cells to assume accurate three-dimensional structures, contributing significantly to mimicking both the form and functionality of the intestine (14). Nikolce’s study illustrates that the inclusion of Laminin in organoid medium enhances cell adhesion to basement membranes (15). Fibrin, formed from fibrinogen as a vital element in blood clotting, effectively replicates the matrix environment observed during coagulation and healing. Its biodegradability renders Fibrin a flexible component in organoid cultures (16, 17). While natural matrices play a crucial role in intestinal organoid culture, their complexity and variability pose challenges. To address these issues, researchers have turned to chemically synthesized substrates. Common synthetic matrices, including PEG-Based Hydrogels, Synthetic Peptide Matrices, Synthetic Polymers, and Self-Assembling Materials, have emerged. Cross-linked polyethylene glycol-based hydrogels, valued for their biocompatibility and degradability, have been employed for mammalian cell cultures. Furthermore, PEG-acrylate monomers, when combined with enzymatically-enhanced hydrogels, have shown promise in cultivating intestinal organoids (15).

**Table 1:** Different types of biomaterials other than Matrigel used for three-dimensional organoid cultures.

BIOMATERIAL	MATERIAL TYPE	CELL TYPE	FEATURES
Alginate	Natural	hPSCs	• The HA template supported the formation of cardiac organoids • Alginate supported differentiation and maturation of the organoids *in vivo*, and they also retained the potential to develop into mature intestinal tissue that resembles human fetal intestine
Chitosan PFC- Chitosan micro gel- for spheroids	Natural	hiPSCs	• hiPSCs cultured on Chitosan membranes could be self-assembled into three- dimensional (3D) spheroids, and be efficiently differentiated into three germ layers. • Easy to observe and convenient to culture • Perflurocarbon modified chitosan prevents hypoxia at the centre of the spheroids
PEG (Polyethylene Glycol) PLGA (Poly Lactic Glycolic Acid) PCL (Poly Caprolactone)	Synthetic Synthetic Synthetic	Lgr5+ ISCs hPSCs Lgr5+ cells HaCaT and Hs68 Cells Breast cancer cells	• Improved colony formation efficiency and Lgr5 expression in PEG gels • Improved growth and expansion of the organoids • Improved wound healing with PLGA nanoparticles loaded into organoids • PCL served as a strong adhesive, preventing intermixing of two cell types • Improved tumoroid formation with porous PCL substrate
Silk	Natural	hESCs hiPSCs	• Cells are highly proliferative and differentiative within silk • High stromal cell infiltration in the silk scaffolds
pNIPAm Collagen gels	Synthetic Natural	Intestinal crypt cells Bmi1+ cells	• Long term culture facilitated, spontaneous villi formation • Mimics natural environment and induces fibroblast differentiation during matrix remodelling
PVA PHEMA Hybrid hydrogels	Synthetic Synthetic Synthetic Gelatin & PEG gel HA & PEG gel	hPSCs βcells MSCs Bone marrow derived Stromal cells	• Formation of kidney micro-organoids into nephron epithelia and stromal cells • Stiffness induced insulin secretion of βcell organoids in PHEMA matrix • Bio-degradable • Supports cell differentiation and proliferation • Low immunogenicity *in vivo*

Reproduced with permission from ref (9). Copyright **©** 2021, Elsevier.

Over the last two decades, a multitude of studies have revealed that the elasticity or stiffness of the extracellular matrix (ECM) significantly influences essential cellular processes such as spreading, growth, proliferation, migration, differentiation, and organoid formation. However, not all types of ECM contribute to this in the same way. Different types of ECMs have different compositions and mechanical properties, which can affect how they interact with cells and influence cellular processes. For example. GelMA, synthesized from methacrylic modification of gelatin, mimics the structure of the extracellular matrix and has been widely used as a general-purpose multi-reaction scaffold, including organoid culture, but its stiffness and small pore size inhibit its application in 3D culture (9). Mechanistically, cellular interactions with the ECM involve actions such as pulling (typically facilitated by actin-based contraction linked to ECM via integrin-based adhesion) and pushing (often driven by actin polymerization and microtubule dynamics) (18). The mechanical attributes of ECMs play a pivotal role in mediating these interactions, initiating cellular mechanotransduction and influencing cell behavior. Studies highlight pore size and matrix degradation capacity as pivotal regulators of mechanical constraint. In environments with rigid or elastic pores, matrix degradation becomes crucial for cells to surpass constraints and facilitate migration. Conversely, in sufficiently viscoelastic or viscoplastic environments, cells can overcome limitations to enlarge, deposit matrix, morphologically change during diffusion or mitosis, and migrate. Additionally, apart from pore size and degradability, matrix mechanical viscoplasticity serves as another determinant of confinement. These properties are interconnected during cellular matrix remodeling: cellular restructuring of viscoplastic matrices alters pore size, while matrix degradation impacts their viscoelastic traits, and alterations in matrix structure may influence viscoplasticity and degradability. Furthermore, matrix remodeling and deposition tendencies tend to escalate with increasing viscoplasticity, rendering the mechanical microenvironment of cellular response time-dependent and evolving cell-matrix interactions into a dynamic, potentially iterative process (19).

Nonetheless, the ECM, being a dynamic environmental component, poses challenges in terms of manipulation in both *in vivo* and *in vitro* models (20, 21). Cultures of gut, liver, and pancreas organoids heavily rely on 3D hydrogel matrices. While these culture systems are essential for supporting stem cell growth, they also face limitations in their ability to manipulate the extracellular matrix (22–24).To address the aforementioned issues, researchers have employed chemical or enzymatic cross-linking techniques to tether signaling proteins within natural ECMs. This approach allows the study of the diverse components within ECMs by altering their biochemical and physical attributes. For instance, scientists have incorporated intact molecules or peptides into synthetic hydrogels for 3D culture (20, 25). Presently employed peptides encompass the RGD peptide, the collagen-derived peptide GFQGER, and the laminin-derived peptides IKVAV and YIGSR. Furthermore, researchers have engineered biomaterials designed for matrix remodeling and degradation. These materials encourage cell-mediated degradation by incorporating multiple MMP-sensitive amino acid sequences, specifically tailored for use in aqueous gels as cross-linking agents (15, 26). ECM degradation can also be achieved independently. Synthetic hydrogels with ester groups in their structure can undergo hydrolysis gradually over time. In contrast to hydrolysis and ester degradation, photodegradation can be meticulously controlled both in terms of timing and spatial precision (27–29).

While the extracellular matrix (ECM) plays a crucial role in cultivating intestinal tumor organoids, the most pivotal aspect of intestinal tumor organoid culture lies in the formulation of tumor organoid induction media (30, 31). The induction factors vary for different types of intestinal tumors, and the development of organoid culture media is particularly challenging due to the personalized nature of individual tumor patients. Nevertheless, certain components remain constant, with key inducible cytokines primarily including Noggin (32), R-Spondin (33), and EGF (34), and inducible small molecules mainly consisting of Y-27632 (35) and N-acetyl-L-Cysteine (36). Despite the complexity associated with the individualization of culture conditions, the standardization of some components provides a foundation for advancing the field of intestinal tumor organoid culture.

## 3 Origin and development of the intestinal organoids

Intestinal organoids can, to a certain extent, mirror the structure and function of native intestinal tissues, primarily stemming from pluripotent stem cells (PSCs) and intestinal stem cells (ISCs). As a result, there are three primary categories of intestinal organoids: epithelial organoids derived from ISCs, multicellular organoids derived from PSCs, and multicellular organoids derived from tissues (37, 38). Epithelial crypts are widely acknowledged to maintain proximity with subepithelial myofibroblasts, with a prevailing belief that these cells establish a distinct and specialized cellular niche at the bottoms of the crypts (39). Conventionally, stem cell niches are depicted as pre-existing locations where stem cells migrate. However, Sato et al. presented a novel perspective, revealing that intestinal stem cells receive crucial niche support from their own specialized progeny, specifically the Paneth cells. These Paneth cells assume a multifaceted role as protectors of stem cells, not only by releasing substances that fight bacteria but also by providing indispensable signals essential for the niche. Their study showcased that Wnt3-producing Paneth cells, in conjunction with soluble factors like EGF, R-spondin, and noggin, can function as a basic *in vitro* niche for intestinal stem cells (40). An intriguing observation arises while Wnt secretion by Paneth cells is essential for preserving Lgr5 stem cells *in vitro*, it appears nonessential *in vivo*. Subsequent research has elucidated that neighboring mesenchymal cells act as an alternative supplier of Wnt, both within cultured environments and in living organisms. Remarkably, Wnt secretion by either Paneth cells or the mesenchyme adequately sustains the intestinal stem cell niche *in vivo (* 41–43).

Previously, Sato et al. achieved a significant breakthrough by placing single Lgr5^+^ intestinal stem cells (ISCs) and crypts into a serum-free medium enriched with essential factors including EGF, the Wnt agonist R-spondin, and the BMP inhibitor Noggin, among others. Through co-cultivation, they successfully developed a three-dimensional cystic luminal structure, establishing intestinal epithelial tissues that closely resembled their *in vivo* counterparts and maintained the same functionality. This groundbreaking work marked the inception of the mouse intestinal organoid culture system featuring a crypt-villus structure, commonly referred to as the “ENR system,” and has since ushered in a new era in organoid research. Simultaneously, Sato et al. discovered that the incorporation of recombinant Wnt3A into the ENR medium enabled the cultivation of mouse colon organoids. They further fine-tuned the culture methodology for human small intestine and colon organoids, rooted in the ENR system, which also proved applicable for culturing Apc-deficient adenomas in mice and tumor organoids in human colorectal cancer cells. This achievement in establishing an *in vitro* 3D culture system centered on intestinal stem cells represents a pivotal breakthrough in this domain (39).

Lgr5^+^ is considered a marker gene for the identification of intestinal stem cells (6). Adult intestinal stem cells display *in vivo* proliferation and long-term survival, which has enabled researchers to successfully isolate individual Lgr5^+^ intestinal stem cells and cultivate them *in vitro*, serving as a wellspring for intestinal organoids. Typically, intestinal organoids cultured from ISCs adopt a spherical morphology and encompass a diverse array of functional epithelial cells, including Lgr5^+^ stem cells, Paneth cells, goblet cells, and enterocytes (39, 44). Research has demonstrated that *in vitro*-cultured intestinal organoids can be sustained for over six months when subjected to suitable environmental conditions. Thus far, both mouse and human intestinal organoids have been cultivated successfully (45). Intestinal epithelial homeostasis primarily hinges on Lgr5^+^ crypt basal columnar cells (Lgr5^+^CBC), the overwhelming majority of which originate from Lgr5^+^CBC. Chronic injuries and inflammatory responses within the intestinal epithelium can trigger the intrinsic immune response of the intestinal mucosa, thereby altering the local microenvironment of the intestinal lumen and ultimately leading to the restructuring of intestinal tissues. In a homeostatic environment, intestinal crypt stem cells (ICS) alone are insufficient to replace damaged tissue and are less conducive to the regenerative repair of the intestinal epithelium. This underscores the need for *in vitro* simulation of the intestinal epithelial injury repair process, which relies on ISC—an effective process capable of responding to injury (6). Qu et al. present a groundbreaking organoid culture method centered around novel small intestinal organoids possessing regenerative capabilities for injury repair. They further introduce a novel combination of epithelial chemical small molecules designed to facilitate the restoration of intestinal epithelial tissue in a mouse model (46). This culture condition comprises eight chemical components designed to amplify the expression of regenerative signals associated with injury repair while preserving the expression of gut-specific markers. Hence, it is referred to as the 8C culture system. In contrast to traditional ENR organoid culture, these newly developed organoids exhibit a significantly enriched damage-responsive stem cell population characterized by a more intricate villous structure, accelerated growth, and a higher organoid formation rate. Furthermore, they demonstrate a substantially enhanced expansion capacity, facilitating long-term passaging and genome stability. These novel cultivation conditions triggered the expression of genes related to injury regeneration during the cultivation of small intestinal organoids. This stimulation resulted in the development of more intricate structures and offered fresh *in vitro* models for investigating epithelial tissue regeneration.

Certain matrix components can supply the necessary ligands and varied concentrations of growth factors that mimic physiological conditions, thereby preserving the properties of stem cells. However, *in vitro* culture models for intestinal stem cells often lack these essential matrix components (47). Consequently, researchers have devised two more intricate intestinal organoid systems: multicellular organoids derived from PSCs and multicellular organoids originating from tissue, encompassing a range of cell types such as mesenchymal cells, enteric neuronal cells, vascular cells, and immune cells (48–50). Spence et al. accomplished the generation of sophisticated intestinal organoids by initiating developmental processes in pluripotent stem cells (PSCs). Subsequently, adult colonic organoids were cultivated by fine-tuning two signaling pathways, bmp and wnt. The organoids produced through this approach exhibited an array of epithelial and mesenchymal cells, closely resembling the development of the infant intestine (51–53).

## 4 Advancements in intestinal tumor organoid research

Colorectal cancer is the third most diagnosed cancer and the second leading cause of cancer-related mortality worldwide. Patient-derived organoids (PDOs) for colorectal cancer have been effectively generated from diverse histologic subtypes, including rare ones such as neuroendocrine and mucinous carcinomas. These PDOs have been successfully cultured from both tumor resection specimens and biopsy samples, achieving success rates ranging from 60% to 100% (54, 55). Presently, the cultivation of intestinal tumor-like organs follows a process similar to that for normal intestinal-like organs, involving the digestion of tumor tissues to isolate tumor cells, followed by their culture in a matrix gel. Nevertheless, unlike the procedure for normal intestinal-like organs, intestinal adenocarcinoma-like organs harbor multiple sustained activation mutations in oncogenes, enabling their survival in a medium devoid of Wnt and R-spondin1. As a result, a selective growth factor approach can be employed to specifically cultivate intestinal adenocarcinoma-like organs (44). *In vitro* construction of intestinal cancer organoids using intestinal adenoma or adenocarcinoma tissues can retain the genetic stability and diversity characteristic of the primary tumor. This novel approach presents a valuable model for investigating the intricacies of colorectal carcinogenesis, disease progression, and tailoring individualized anticancer therapies (56, 57).

Cultures of patient-derived cancer organoids represent a vital approach for mirroring clinical heterogeneity. Throughout tumorigenesis and progression, there is often aberrant activation of pertinent signaling pathways or irregular expression of growth factors linked to these pathways. While these growth factors are not intrinsic to the culture medium, their irregular expression can impact tumor differentiation. Fujii et al. established a colorectal cancer PDO library encompassing 55 PDOs representing diverse histologic subtypes and clinical stages. Their research revealed that the proliferation of colorectal cancer PDOs was influenced by factors such as Wnt3A, R-spondin1, SB202190, and oxygen concentration (54). The histological, genomic, transcriptomic, and proteomic features of intestinal organoids cultivated using this method closely mirrored those of the original tumors. Schumacher et al. additionally confirmed that colon carcinoma organoids exhibited the same markers as the primary tumors (58, 59). Hence, researchers can establish a model of intestinal tumor organoids through the manipulation of elements in the culture medium. Furthermore, the use of CRISPR/Cas9 gene editing technology presents the opportunity to leverage organoids as cancer models. In related studies, there have been instances of sequential CRISPR-induced mutations in APC, TP53, KRAS, and SMAD4 within intestinal organoids (54), Designing specific culture media tailored to each tumor-like organ is of paramount importance. In the case of colon cancer, the characteristic activation of the Wnt signaling pathway can be replicated by excluding Wnt and R-spondin1 from the culture medium. Similarly, for colorectal cancers marked by alterations in the EGF receptor pathway, it is feasible to establish cultures by omitting EGF (60). In the case of certain metastatic colon cancer carcinoids obtainable through this method, it was discovered that around 90% of somatic mutations corresponded between metastatic colorectal cancer carcinoids and the primary tumor tissue. Additionally, a correlation of 0.89 was observed in the DNA copy number between the human-derived carcinoids and their associated tissues (61). Organoids cultivated through this technique preserve the histopathological traits of the primary tumor while upholding genomic stability. Consequently, they serve as a valuable platform for comprehensive investigations into the fundamental aspects of tumors and find applications in clinical translational medical research.

One of the major hurdles in cultivating tumor organoids is the proliferation of normal epithelial organoids during primary culture, and numerous approaches have been developed to tackle this challenge (58). To tackle this obstacle, several strategies can be explored: modifying culture conditions, incorporating targeted inhibitors or growth-promoting factors, and refining the timing and techniques involved in the culturing process. If there’s an overgrowth of normal epithelial cells during the cultivation of tumor-like organs, it could lead to contamination and the loss of tumor cells during passage. To prevent this, meticulous care is crucial during the initial tissue dissection phase to isolate tumor tissue from the surrounding normal tissue during sampling. This minimizes the presence of normal epithelial cells in collected samples. Subsequently, in cultivation, certain chemical agents can be employed to segregate these tissues. For instance, Sato et al. used EDTA chelating buffer to incubate mouse adenoma intestinal fragments on ice for 60 minutes. Following a wash with cold chelating buffer, most normal intestinal epithelial cells were shed, while adenoma cells remained attached to the stroma (44). In organoid culture, the omission of crucial growth factors for normal epithelial cells can hinder their proliferation. Holloway et al. observed endothelial cell populations during the initial phases of pluripotent stem cell-derived intestinal organoid culture, noting a decline in their numbers over extended culture periods. By supplementing the culture medium with cytokines like EGF, VEGF, bFGF, and BMP4, endothelial cells could be stimulated to differentiate and facilitate the formation of vascularized small intestinal organoids (48). In the culture of human colorectal tumor organoids, the inclusion of Wnt3A/R-spondin1 (WR) isn’t necessary when the Wnt signaling pathway is already aberrantly activated by mutations in the APC gene. Similarly, if mutations affect the RAS/MAPK signaling pathway, supplementing with EGF growth factor becomes unnecessary. Additionally, when tumor mutations inhibit the function of the TGF-β signaling pathway, the use of a TGF-β inhibitor (A83-01) is not required to sustain growth (54). Gene editing technology offers a solution to these challenges as well. Matano et al. employed CRISPR-Cas9 gene editing to induce mutations in a range of oncogenes including APC, SMAD4, TP53, KRAS, and PI3K within normal epithelial organoids. They cultured tumorigenic organoids with invasive and metastatic capabilities by utilizing selective media that omitted the specific growth factors associated with the edited oncogenes (62).

## 5 Intestinal organoid and translational research

Translational research encompasses the conversion of findings from basic scientific research into practical clinical applications. Its primary objective is to employ experimental breakthroughs in the diagnosis, treatment, and prevention of diseases (63). Intestinal organoids hold significant relevance in translational research. As colorectal cancer organoid models are explored comprehensively and continue to evolve, they have made a profound impact on our understanding of tumor mechanisms and treatment. Nevertheless, harnessing this knowledge to enhance therapeutic strategies for intestinal tumors poses a significant challenge.

### 5.1 Application of intestinal organoids in drug screening

**Figure 2:**
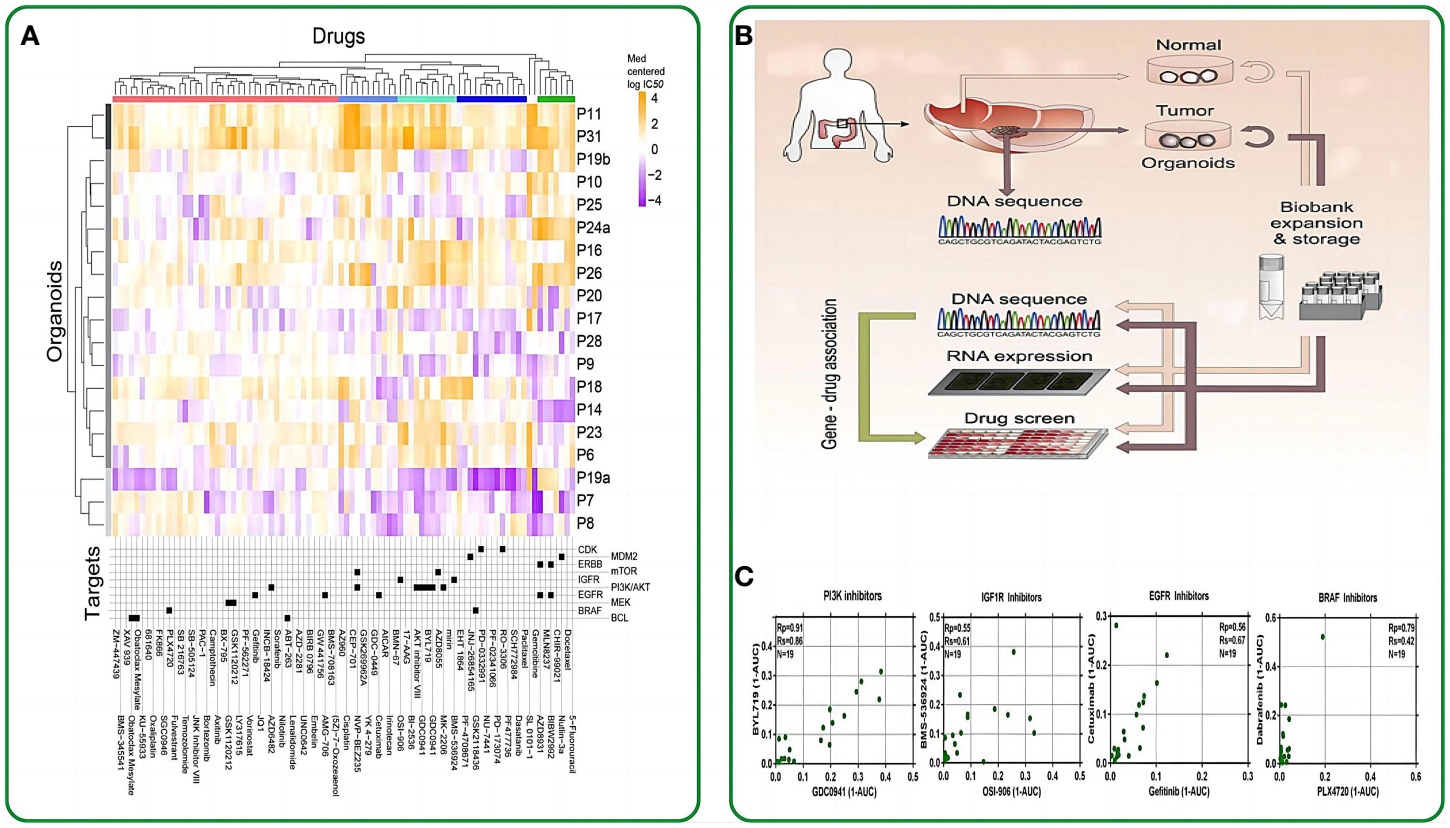
The application of intestinal organoids in drug screening. **(A)** Organoids have been clustered based on their IC50 values across the drug panel. The drug names and their nominal target(s) are provided in the bottom panel. **(B)** Application flowchart of gut like organs in drug screening. **(C)** Drugs with the same nominal targets have similar activity profiles across the organoid panel. (1-AUC) values are plotted for inhibitor of PI3K (GDC0941 and BYL719), IGF1R (OSI-906 and BMS-536924), EGFR (cetuximab and gefitinib), and BRAF (PLX4720 and dabrafenib). Reproduced with permission from ref (58). Copyright **©** 2015, Elsevier.

Traditional 2D cell lines are susceptible to mutations during culture or potential contamination from the culture medium and other cell lines, thereby constraining their utility in disease prediction and drug screening applications (**Figure 2**) (64, 65). Patient-derived intestinal organoids offer a partial solution to this issue. By sourcing intestinal tumor tissue from patients and cultivating intestinal tumor organoids *in vitro*, it becomes possible to conduct more comprehensive mutational and phenotypic analyses.

Clevers et al. made a pioneering move by introducing intestinal organoids into 384-well plates and subjecting them to a panel of 83 drugs. These drugs included 10 chemotherapeutic drugs, 25 clinically utilized drugs, 29 drugs in clinical trials, and 29 targeted drugs. Their study unveiled a notable correlation between the genotypes of the patient-derived organoids (PDOs) and their responses to these drugs. For instance, TP53-mutated intestinal tumor organoids exhibited remarkable resistance to MDMI inhibitors, while KRAS-mutated organoids displayed extreme resistance to ERBB inhibitors (58). This groundbreaking study marked the initial application of drug screening on intestinal organoids, underlining the potential of these organoids as pertinent biomarkers for therapeutic response assessments. In the work of Vlachogiannis et al., drugs from phase 1-3 clinical trials or already in clinical use were administered to a metastatic colon cancer organoid model. This approach enabled the screening of drugs suitable for patients with colon cancer. Consequently, several genotypes were found to correlate with drug phenotypes. For example, an ERBB2-amplified intestinal tumor-like organoid exhibited significant responsiveness to the dual ERBB2/EGFR inhibitor lapatinib, but not to a strain with an epidermal growth factor receptor-amplified status with a normal ERBB2 allele (59). On October 2020, the University of Basel and Novartis Institutes for Biomedical Research conducted a groundbreaking study using intestinal organoids for high-throughput drug screening. This research aimed to identify organoid phenotypic profiles and explore the impact of RXR receptor inhibitory drugs on their regulation. The study revealed functional gene interaction profiles governing organoid development and their regenerative potential. The researchers utilized 15-element fingerprint clusters to categorize compound-treated organoids and demonstrated that a multivariate phenotypic screening approach can uncover the complex mechanisms of intestinal regeneration (66).

Intestinal organoids present a realistic tumor model. Leveraging these organoids, researchers can assess the effectiveness and toxicity of various drugs against tumors, facilitating the screening of more potent antitumor medications. This approach expedites the drug development process and eliminates the necessity for animal models or direct experimentation on patients.

### 5.2 Personalized therapy with intestinal organoids

Personalized therapy involves devising a targeted treatment strategy based on individual patient distinctions and specific tumor characteristics. The development of intestinal tumor organoids allows for the utilization of a patient’s tumor samples, thus enabling the tailoring of personalized treatment plans for each patient. By examining how a patient’s tumor responds to different drugs within these organoids, medical professionals can enhance their ability to forecast treatment effectiveness and select the most suitable treatment regimen. Numerous studies have, thus far, harnessed PDO biobanks for customized drug screening in patients with various intestinal tumors. In 2019, a groundbreaking I/II clinical trial marked the initial demonstration of the value of PDOs in personalized medicine, employing PDO biobanks derived from patients with metastatic colon and gastroesophageal cancers (59). Another study provided further evidence of the substantial potential of PDOs as a preclinical tool for predicting patient outcomes with intestinal tumors. In this study, human-derived intestinal tumor carcinoids were introduced into immunodeficient mice, resulting in the development of aggressive colon cancers. The administration of chemotherapeutic agents, 5-fluorouracil and oxaliplatin, triggered apoptosis in the tumor carcinoids. This experiment revealed variations in the response of the transplanted tumors to chemotherapy, underscoring the predictive power of PDOs for patient outcomes (67). In contrast, in an *in vitro* drug sensitivity test, PDO was able to predict prognosis in 80% of patients receiving irinotecan but not in those receiving 5-fluorouracil plus oxaliplatin therapy (68). Yao et al. extended this approach by applying neoadjuvant radiotherapy (comprising radiotherapy, 5-fluorouracil, or irinotecan) to assess its effectiveness. They utilized a model involving 96 colon cancer-like organoids from 80 patients. The outcomes of this study revealed a strong correlation between the chemotherapeutic response of PDOs and the real response exhibited by the corresponding patients. The accuracy stood at 84.43%, with a sensitivity of 78.01% and a specificity of 91.97% (69). Collectively, these findings imply that PDOs hold the potential to forecast a patient’s prognosis and facilitate the customization of radiotherapy or chemotherapy protocols with the use of suitable drugs. This approach aims to mitigate the adverse effects of radiotherapy on patients and enhance their overall quality of care.

From the current standpoint, the utilization of intestinal tumor organoids in personalized medicine holds the promise of enhancing therapeutic effectiveness, mitigating unnecessary treatment side effects, and delivering patients with highly individualized and precise treatment strategies. Nevertheless, achieving personalized treatment necessitates robust genetic testing, comprehensive clinical data support, and a cohesive partnership among medical teams.

## 6 Limitations and future prospects

Over the past decade, organoid technology has witnessed remarkable advancements in the exploration of intestinal tumors. The establishment of organoid cultures for various tumor types has wielded a profound influence on tumor-related research, significantly contributing to disease modeling and drug screening. Tumor organoids effectively serve as avatars of a patient’s tumor, empowering clinicians to anticipate responses to anticancer medications and formulate individualized therapeutic strategies.

Challenges persist in intestinal tumor organoid culture, including the absence of a microenvironment and the functional vascular, nervous, or immune systems, which exist in animal models but are lacking in organoids. These limitations hinder organoids from replicating all aspects of tumor development *in vivo*, including the preservation of heterogeneity. The most crucial biological attribute of tumors is their heterogeneity, a trait intimately linked to drug resistance and recurrent metastasis in cancer patients. These shortcomings render organoids less adept than *in vivo* models. Consequently, at this stage, animal models continue to be indispensable for vaccine and drug development. Nevertheless, the field is swiftly advancing, with the integration of cells associated with the tumor microenvironment, such as fibroblasts and immune cells, into organoid studies (70, 71).

Future research will focus on the following key aspects: (1) standardizing the culture environment for various organoid types, streamlining time-consuming experiments; (2) ensuring the alignment between organoids and primary tumors at both the pathological and genomic levels; (3) minimizing the cost associated with organoid culture. Artificial organoids will play a crucial role in replicating intricate human organs and simulating intra-organ interactions to investigate the mechanisms underlying disease onset. These organoids, whether sourced from a healthy individual or a patient, will offer a dependable means to evaluate the molecular impacts of susceptibility in individuals of diverse age groups, genders, races, and other categories. As a result, they will pave the way for personalized therapeutic strategies, both in the present and in the future.

In summary, the utilization of intestinal tumor organoids in translational research offers a more authentic and immediate platform for oncology studies. It expedites the advancement of therapeutic approaches, enhances treatment results, and contributes to the realization of personalized medicine.

## Author contributions

TL: Conceptualization, Methodology, Writing – original draft. YH: Conceptualization, Writing – original draft. XL: Conceptualization, Data curation, Methodology, Software, Writing – original draft. HL: Data curation, Formal analysis, Writing – review & editing. JQ: Visualization, Writing – original draft. HX: Investigation, Writing – original draft. JW: Data curation, Methodology, Supervision, Writing – original draft. YH: Funding acquisition, Resources, Writing – review & editing, Conceptualization, Writing – original draft. CZ: Funding acquisition, Resources, Writing – review & editing, Conceptualization, Writing – original draft.
